# Association of Traumatic Brain Injury Severity and Self-Reported Neuropsychiatric Symptoms in Wounded Military Service Members

**DOI:** 10.1089/neur.2022.0063

**Published:** 2023-01-10

**Authors:** Sharon Y. Kim, Alyssa A. Soumoff, Sorana Raiciulescu, Patricia A. Kemezis, Elizabeth A. Spinks, David L. Brody, Vincent F. Capaldi, Robert J. Ursano, David M. Benedek, Kwang H. Choi

**Affiliations:** ^1^ Program in Neuroscience, Uniformed Services University, Bethesda, Maryland, USA.; ^2^ Department of Psychiatry, Uniformed Services University, Bethesda, Maryland, USA.; ^3^ Behavioral Health Directorate, Walter Reed National Military Medical Center, Bethesda, Maryland, USA.; ^4^ Department of Preventive Medicine and Biostatistics, Biostatistics Consulting Center, Uniformed Services University, Bethesda, Maryland, USA.; ^5^ Center for Neuroscience and Regenerative Medicine, Uniformed Services University, Bethesda, Maryland, USA.; ^6^ Department of Neurology, Uniformed Services University, Bethesda, Maryland, USA.; ^7^ Center for the Study of Traumatic Stress, Uniformed Services University, Bethesda, Maryland, USA.

**Keywords:** combat exposure stress, loss of consciousness (LOC), major depressive disorder (MDD), post-traumatic stress disorder (PTSD), somatic symptom disorder (SSD), traumatic brain injury (TBI)

## Abstract

The impact of traumatic brain injury (TBI) severity and loss of consciousness (LOC) on the development of neuropsychiatric symptoms was studied in injured service members (SMs; *n* = 1278) evacuated from combat settings between 2003 and 2012. TBI diagnoses of mild TBI (mTBI) or moderate-to-severe TBI (MS-TBI) along with LOC status were identified using International Classification of Diseases, Ninth Revision (ICD-9) codes and the Defense and Veterans Brain Injury Center Standard Surveillance Case Definition for TBI. Self-reported psychiatric symptoms were evaluated for post-traumatic stress disorder (PTSD) with the PTSD Checklist, Civilian Version for PTSD, the Patient Health Questionnaire-9 for major depressive disorder (MDD), and the Patient Health Questionnaire-15 for somatic symptom disorder (SSD) in two time periods post-injury: Assessment Period 1 (AP1, 0.0–2.5 months) and Assessment Period 2 (AP2, 3–12 months). mTBI, but not MS-TBI, was associated with increased neuropsychiatric symptoms: PTSD in AP1 and AP2; MDD in AP1; and SSD in AP2. A subgroup analysis of mTBI with and without LOC revealed that mTBI with LOC, but not mTBI without LOC, was associated with increased symptoms as compared to non-TBI: PTSD in AP1 and AP2; MDD in AP1; and SSD in AP1 and AP2. Moreover, mTBI with LOC was associated with increased MDD symptoms in AP2, and SSD symptoms in AP1 and AP2, compared to mTBI without LOC. These findings reinforce the need for the accurate characterization of TBI severity and a multi-disciplinary approach to address the devastating impacts of TBI in injured SMs.

## Introduction

Traumatic brain injury (TBI) and its long-term neuropsychiatric sequelae garnered significant public attention in the past decade, particularly within military populations.^1–5^ According to the Department of Defense (DoD) evaluation of U.S. military casualty statistics, almost 450,000 U.S. military personnel worldwide experienced a TBI between 2000 and 2021: 82.3% were mild, 10.7% moderate, and 1.0% severe.^6^ However, the proportion of moderate and severe TBI is substantially higher among military personnel exposed to combat.^7,8^ Survival rates of patients with moderate-to-severe TBI (MS-TBI) have improved, highlighting the need to understand long-term neuropsychiatric consequences.^9,10^ Likewise, mild TBI (mTBI), once deemed a relatively benign injury, has been increasingly recognized as a major cause of adverse outcomes.^11–15^ Persons with TBI, including mTBI, frequently experience persistent neuropsychiatric symptoms, including chronic pain, anxiety, irritability, depression, headaches, cognitive impairment, and sensory deficits.^16–21^

The precise role of TBI severity on differential neuropsychiatric outcomes and symptom onset remains ambiguous. Previous studies have proposed that there is a “dose-response relationship” between TBI severity and neuropsychiatric outcomes, in which greater TBI severity (moderate or severe) is proportional to worse long-term neurocognitive and -behavioral symptoms.^22–26^ However, this notion is disputed by other studies that have found an inverse relationship between TBI severity and symptom severity, in which those with mTBI exhibit more symptoms than those with MS-TBI.^10,27–29^ Interestingly, other studies did not find associations between TBI severity and neuropsychiatric outcomes, including measures of grief, fatigue, headaches, pain, and cognitive complaints.^30,31^ Additionally, various severities of TBI may lead to different onset of neuropsychiatric symptom manifestation, with previous studies reporting that MS-TBI leads to delayed symptom onset as compared to mTBI in both military^32–34^ and civilian^20,35,36^ populations.

Ambiguity regarding TBI severity raises the question of the potential role of loss of consciousness (LOC) on neuropsychiatric sequelae. According to the Veterans Affairs and DoD clinical practice guidelines for the management of concussion, TBI severity can be graded by duration of LOC, in which mTBI is defined as a concussion with LOC <30 min and MS-TBI with LOC >30 min.^2^ It has been suggested that LOC may be protective against post-traumatic stress disorder (PTSD) development attributable to amnesia and lack of memory of the traumatic event.^37–39^ However, recent studies, especially those considering data from the conflicts in Iraq and Afghanistan, have demonstrated that TBI, even those with LOC, is an important risk factor for PTSD and other psychiatric outcomes.^40,41^ Yet, given other evidence that mTBI carries a higher risk of PTSD than more severe TBI,^42^ it remains possible that extended LOC and amnesia present in severe TBI (as opposed to mTBI) could exert protective effects on neuropsychiatric sequelae. Whether this phenomenon is related to certain aspects of traumatic memory consolidation and mediated by LOC or other factors remains to be determined.

The purpose of the current study was to 1) determine the association of TBI severity and self-reported neuropsychiatric symptoms related to PTSD, major depressive disorder (MDD), and somatic symptom disorder (SSD) and 2) examine neuropsychiatric outcomes of TBI patients based on LOC status. We hypothesized that mTBI may lead to greater symptom severity compared to MS-TBI, and mTBI with LOC may contribute to increased self-reported symptoms.

## Methods

### Subject characteristics

The study sites included Walter Reed Army Medical Center (2003–2011) and Walter Reed National Military Medical Center (2011–2012). Study subjects were hospitalized service members (SMs) medically evacuated from Iraq and Afghanistan because of their need for continued medical or surgical inpatient treatment. They typically arrived within a few days post-injury and within 72 h of arrival or when medically stable received an initial behavioral health assessment that included self-reported psychiatric screening with standardized assessments.^43^ They were administered similar follow-up assessments in the subsequent months. Medical diagnoses were derived from International Classification of Diseases, Ninth Revision (ICD-9) codes as part of routine care. Permission to analyze data from these deidentified subjects was provided by the institutional review board at the Walter Reed National Military Medical Center, and the study was deemed exempt.

To determine longitudinal effects of TBI severity on neuropsychiatric symptoms, two post-injury time periods were pre-determined as Assessment Periods (APs) based on the distribution of when the behavioral health assessments had been completed. The first AP included the first 75 days post-injury, referred to as Assessment Period 1 (AP1, 0.0–2.5 months), and the second AP occurred between post-injury days 90 and 365 (AP2, 3–12 months). A total of 1278 wounded SMs with ICD-9 codes completed the behavioral health assessments in both AP1 and AP2. Table 1 presents these subjects' characteristics.

**Table 1.** Subject Characteristics

	All subjects (*n* = 1278)	No TBI (*n* = 785)	Mild TBI (*n* = 157)	Moderate-to-severe TBI (*n* = 336)
Continuous variables	Mean (SD)			
Age (years)	27.4 (6.9)	27.4 (7.0)	27.5 (6.4)	27.5 (6.9)
CESS (maximum possible score: 11)	6.3 (2.7); *n* = 1271	6.2 (2.7); *n* = 782	6.6 (2.6); *n* = 156	6.3 (2.7); *n* = 333

Categorical variables	*N* (%)			
Sex (male)	1248 (97.7)	766 (97.6)	154 (97.7)	328 (97.6)
				
Education				
High school or less	615 (48.7)	366 (47.1)	79 (51.0)	170 (51.2)
Some college or more	649 (51.3)	411 (52.9)	76 (49.0)	162 (48.8)
				
Marital status				
Married	616 (48.2)	371 (47.3)	88 (56.1)	157 (46.9)
Single/separated/divorced^a^	661 (51.8)	414 (52.7)	69 (43.9)	178 (53.1)
				
Branch				
Army	1214 (95.1)	745 (95.0)	149 (95.5)	320 (95.2)
Air Force/Marine Corps/Navy^a^	62 (4.9)	39 (5.0)	7 (4.5)	16 (4.8)
				
Military rank				
Enlisted (E1–E9)	1164 (91.5)	717 (91.8)	141 (90.4)	306 (91.3)
Officers (O1+) or warrant officers	108 (8.5)	64 (8.2)	15 (9.6)	29 (8.7)
				
Duty status				
Active duty	951 (74.4)	561 (71.5)	124 (79.0)	266 (79.2)
Reservist or retired^a^	327 (25.6)	224 (28.5)	33 (21.0)	70 (20.8)
				
No. of deployments				
1	667 (52.6)	405 (52.0)	78 (50.6)	184 (55.1)
2 or more	600 (47.4)	374 (48.0)	76 (49.4)	150 (44.9)
				
Injury Severity Score				
<16	693 (54.2)	507 (64.6)	88 (56.1)	98 (29.2)
≥16	585 (45.8)	278 (35.4)	69 (43.9)	238 (70.8)
				

Percentages of valid answers.

^a^ Referred to as “other” in the article text.

CESS, Combat Exposure Severity Score; SD, standard deviation; TBI, traumatic brain injury.

### Severity of traumatic brain injury

The Defense and Veterans Brain Injury Center (DVBIC), Armed Forces Health Surveillance Branch, and the Centers for Disease Control and Prevention have collaborated to develop a standard TBI surveillance case definition using ICD-9 codes.^44^ TBI diagnoses were identified, and the severity of each TBI was based on the most severe injury charted and classified as mild or moderate-to-severe using the DVBIC 2015 criteria (Supplementary Data S1), similar to other studies.^45,46^ mTBI subjects were further classified as mTBI with LOC and mTBI without LOC for the subgroup analysis.

### Neuropsychiatric symptom assessments

Neuropsychiatric assessments included three distinct self-report questionnaires, including the PTSD Checklist, Civilian Version (PCL-C), Patient Health Questionnaire-9 for MDD, and Patient Health Questionnaire-15 for SSD. All three screening tools were utilized at AP1 and AP2 (Supplementary Data S2) for all participants. Because past trauma influences psychiatric outcomes,^47^ we incorporated combat trauma exposure into the analysis. SMs completed a survey containing 11 potentially stressful combat experiences during the deployments. Total Combat Exposure Severity Score (CESS) for each subject was computed by totaling the tally of positive responses for all 11 questions (Supplementary Data S3) with a range of total scores of 0–11, similar to a previous study.^48^

### Physical injury assessment

Physical injuries were assessed with the Abbreviated Injury Scale (AIS) and Injury Severity Score (ISS), which utilize clinician-rated scales to grade the severity of physical injury in patients.^49^ The ISS is a composite score derived from AIS values from six body regions and is considered the gold standard of injury severity in PTSD studies of civilian and military populations.^50,51^ For each SM, AIS values were determined by clinicians based on injury severity at onset and did not change over time. Severe injury was defined as ISS ≥16, like other studies.^52–56^

### Statistical analysis

All data were analyzed using the Statistical Package for the Social Sciences (SPSS; version 25; SPSS, Inc., Chicago, IL). Binary logistic regression models were used to assess relationships between the presence of self-reported PTSD, MDD, and SSD at AP1 and AP2 with TBI severity (mTBI or MS-TBI). In the first model, the odds of developing probable PTSD, MDD, and SSD after mTBI and MS-TBI were calculated relative to the no-TBI reference group. In a subgroup analysis within the same model, the odds of developing probable PTSD, MDD, and SSD after mTBI without and with LOC were calculated relative to the no-TBI reference group. In a second model, the odds of developing probable PTSD, MDD, and SSD after mTBI with LOC and MS-TBI were calculated relative to the mTBI without LOC reference group. All subjects' demographic variables were included in the multiple logistic regression models, unless otherwise noted. Subjects' characteristics are shown in Table 1 with more details in Supplementary Data S2.

## Results

Of the entire cohort (*n* = 1278) of subjects, 39% were diagnosed with TBI (*n* = 493), whereas 12.3% and 26% were diagnosed with mTBI (*n* = 157) and MS-TBI (*n* = 336), respectively (Table 1). The majority of the entire cohort were male (97.7%), enlisted (91.5%), active duty (74.4%), and in the army (95.1%). Also, 51.3% had education beyond high school, 48.2% were married, and 47.4% had two or more deployments. Mean CESS was 6.3 (standard deviation [SD] = 2.7) for the entire cohort and was not found to be significantly different between TBI groups. The percentage of SMs with a high ISS (≥16) was 45.8% in the entire cohort, 35.4% in the non-TBI group, 43.9% in the mTBI group, and 70.8% in the MS-TBI group.

In the early period post-injury (AP1), rates of PTSD (225%) and MDD (281%) were higher in the mTBI group whereas rates of PTSD (58%) and MDD (55%) were lower in the MS-TBI group, as compared to the no-TBI group. However, in the late period post-injury (AP2), rates of PTSD were higher in both the mTBI (183%) and MS-TBI groups (125%), as compared to the no-TBI group (Table 2).

**Table 2.** Self-Reported PTSD, MDD, and SSD in All Subjects

	All subjects (*n* = 1278)	No TBI (*n* = 785)	Mild TBI (*n* = 157)	Moderate-to-severe TBI (*n* = 336)
AP1 positive screen: *n* (%)				
PTSD	41 (3.2)	24 (3.1)	11 (7.0)	6 (1.8)
MDD	38 (3.0)	21 (2.7)	12 (7.6)	5 (1.5)
SSD	91 (7.1)	55 (7.0)	13 (8.3)	23 (6.8)
AP2 positive screen: *n* (%)				
PTSD	145 (11.3)	76 (9.7)	28 (17.8)	41 (12.2)
MDD	95 (7.4)	55 (7.0)	12 (7.6)	28 (8.3)
SSD	88 (6.9)	47 (6.0)	18 (11.5)	23 (6.8)

Percentages of valid answers.

AP, assessment period; MDD, major depressive disorder; PTSD, post-traumatic stress disorder; SSD, somatic symptom disorder; TBI, traumatic brain injury.

Rates of probable PTSD, MDD, and SSD among TBI subjects with and without LOC are delineated in Table 3. Compared to mTBI without LOC, mTBI with LOC showed increased rates of PTSD (265%), MDD (198%), and SSD (534%) in the early period post-injury (AP1). Similarly, in AP2, mTBI with LOC was associated with higher rates of PTSD (152%), MDD (488%), and SSD (257%) than mTBI without LOC.

**Table 3.** Self-Reported PTSD, MDD, and SSD in mTBI Subjects With and Without LOC

	Mild TBI without LOC (*n* = 78)	Mild TBI with LOC (*n* = 79)
	AP1 positive screen: *n* (%)	
PTSD	3 (3.8)	8 (10.1)
MDD	4 (5.1)	8 (10.1)
SSD	2 (2.6)	11 (13.9)
	AP2 positive screen: *n* (%)	
PTSD	11 (14.1)	17 (21.5)
MDD	2 (2.6)	10 (12.7)
SSD	5 (6.4)	13 (16.5)

Percentages of valid answers.

AP, assessment period; LOC, loss of consciousness; MDD, major depressive disorder; mTBI, mild TBI; PTSD, post-traumatic stress disorder; SSD, somatic symptom disorder; TBI, traumatic brain injury.

Results of the logistic regression analyses for the mTBI and MS-TBI groups are summarized in Table 4. Compared to the non-TBI group, mTBI was associated with increased PTSD in both AP1 (adjusted odds ratio [aOR], 2.34; 95% confidence interval [CI], 1.11–4.96; *p* < 0.05) and AP2 (aOR, 2.02; 95% CI, 1.24–3.30; *p* < 0.01), MDD in AP1 (aOR, 3.42; 95% CI, 1.60–7.33; *p* < 0.01), and SSD in AP2 (aOR, 2.03; 95% CI, 1.13–3.65; *p* < 0.05). However, MS-TBI was not significantly associated with increased rates of PTSD, MDD, or SSD in either AP1 or AP2.

**Table 4.** Unadjusted and Adjusted Risk of Self-Reported PTSD, MDD, and SSD by TBI Severity (*n* = 1278)

	Mild TBI (*n* = 157)		Moderate-to-severe TBI (*n* = 336)	
	Odds ratio (95% CI)			
	Unadjusted	Adjusted	Unadjusted	Adjusted
PTSD				
AP1	2.31^*^ (1.15–4.98)	2.34^*^ (1.11–4.96) ^a,1^	0.58 (0.23–1.42)	0.48 (0.18–1.31)^a,1^
AP2	2.03^**^ (1.26–3.25)	2.02^**^ (1.24–3.30) ^2,3,4^	1.30 (0.87–1.94)	1.42 (0.92–2.19)^2,3,4^
MDD				
AP1	3.01^**^ (1.45–6.26)	3.42^**^ (1.60–7.33) ^a,5,6^	0.55 (0.21–1.47)	0.68 (0.24–1.92)^a,5,6^
AP2	1.10 (0.57–2.10)	0.98 (0.49–1.94)^7,8,9^	1.21 (0.75–1.94)	1.31 (0.79–2.16)^7,8,9^
SSD				
AP1	1.20 (0.64–2.25)	1.22 (0.65–2.32)^10,11,12^	0.98 (0.59–1.62)	0.96 (0.56–1.64)^10,11,12^
AP2	2.03^*^ (1.15–3.61)	2.03^*^ (1.13–3.65) ^13,14^	1.15 (0.69–1.93)	0.94 (0.54–1.63)^13,14^

Reference group: no TBI (*n* = 785).

Adjusted models include age, sex, CESS, military rank, marital status, number of past deployments, branch, education, duty status, and Injury Severity Score as covariates.

^a^ Branch not included.

^*^ *p < 0*.05, ^**^p < 0.01, ^***^p < 0.001.

Demographic variables significant in each model:

^1^ CESS (*p* = 0.02), OR 1.18 (1.03–1.36).

^2^ Rank (*p* = 0.002), OR 9.56 (2.28, 40.06), ref: Officers.

^3^ Age (*p* = 0.01), OR 1.04 (1.01, 1.07).

^4^ CESS (*p* = 0.001), OR 1.13 (1.05, 1.22).

^5^ CESS (*p* = 0.001), OR 1.31 (1.11–1.55).

^6^ Sex (*p* = 0.04), OR 0.19 (0.04–0.92), ref: Female.

^7^ Marital status (*p* = 0.01), OR 1.88 (1.16–3.05), ref: Single/Separated/Divorced.

^8^ Rank (*p* = 0.04), OR 2.96 (1.03–8.50), ref: Officer.

^9^ CESS (*p* = 0.03), OR 1.10 (1.01–1.20).

^10^ Education (*p* = 0.04), OR 0.60 (0.37–0.97), ref: Some college or more.

^11^ Sex (*p* = 0.01), OR 0.25 (0.10, 0.66), ref: Female.

^12^ CESS (*p* = 0.02), OR 1.12 (1.02–1.23).

^13^ Rank (*p* = 0.01), OR 6.21 (1.45–26.58), ref: Officers.

^14^ Sex (*p* = 0.002), OR 0.20 (0.08–0.55), ref: Female.

AP, assessment period; CESS, Combat Exposure Severity Score; CI, confidence interval; MDD, major depressive disorder; OR, odds ratio; PTSD, post-traumatic stress disorder; SSD, somatic symptom disorder; TBI, traumatic brain injury.

Table 5 summarizes the association between mTBI with LOC and without LOC, and symptoms. Compared to the non-TBI group, the mTBI with LOC group had increased odds of PTSD in both AP1 (aOR, 3.87; 95% CI, 1.63–9.15; *p* < 0.01) and AP2 (aOR, 2.47; 95% CI, 1.33–4.57; *p* < 0.01). The mTBI with LOC group also had increased odds of MDD in AP1 (aOR, 4.83; 95% CI, 1.99–11.75; *p* < 0.001) and SSD in both AP1 (aOR, 2.39; 95% CI, 1.17–4.87; *p* < 0.05) and AP2 (aOR, 3.14; 95% CI, 1.58–6.23; *p* < 0.001). However, mTBI without LOC was not significantly associated with any of the syndromes in AP1 or AP2.

**Table 5.** Unadjusted and Adjusted Risk of Self-Reported PTSD, MDD, and SSD in mTBI With and Without LOC (*n* = 1278)

	Mild TBI (*n* = 157)			
	Mild TBI without LOC (*n* = 78)		Mild TBI with LOC (*n* = 79)	
	Odds ratio (95% CI)			
	Unadjusted	Adjusted	Unadjusted	Adjusted
PTSD				
AP1	1.27 (0.37–4.31)	1.14 (0.33–3.92)^a,1^	3.57^**^ (1.55–8.25)	3.87^**^ (1.63–9.15) ^a,1^
AP2	1.53 (0.78–3.02)	1.59 (0.79–3.20)^2,3,4^	2.56^**^ (1.42–4.60)	2.47^**^ (1.33–4.57) ^2,3,4^
MDD				
AP1	1.97 (0.66–5.88)	2.16 (0.70–6.65)^a,5,6^	4.10^***^ (1.75–9.59)	4.83^***^ (1.99–11.75) ^a,5,6^
AP2	0.35 (0.08–1.46)	0.33 (0.08–1.37)^7,8,9^	1.92 (0.94–3.94)	1.76 (0.82–3.75)^7,8,9^
SSD				
AP1	0.35 (0.08–1.46)	0.33 (0.08–1.39)^10,11,12^	2.15^*^ (1.07–4.30)	2.39^*^ (1.17–4.87) ^10,11,12^
AP2	1.08 (0.42–2.79)	1.06 (0.40–2.78)^13,14^	3.09^***^ (1.59–6.00)	3.14^***^ (1.58–6.23) ^13,14^

Reference group: no TBI (*n* = 785).

Adjusted models include age, sex, combat stress score, military rank, marital status, number of past deployments, branch, education, and duty status as covariates.

Table 5 is an expansion of Table 4 with the mTBI group separated into two subgroups: 1) mTBI with LOC and 2) mTBI without LOC.

^a^ Branch not included.

^*^ *p < 0*.05, ^**^p < 0.01, ^***^p < 0.001.

Demographic variables significant in each model:

^1^ CESS (*p* = 0.02), OR 1.18 (1.03–1.36).

^2^ Age (*p* = 0.01), OR 1.04 (1.01–1.07).

^3^ Rank (*p* = 0.002), OR 9.52 (2.27–39.89), ref: Officers.

^4^ CESS (*p* = 0.001), OR 1.14 (1.05–1.22).

^5^ CESS (*p* = 0.001), OR 1.31 (1.11–1.55).

^6^ Sex (*p* = 0.04), OR 0.19 (0.04–0.93), ref: Female.

^7^ Marital status (*p* = 0.01), OR 1.90 (1.17–3.08), ref: Single/Separated/Divorced.

^8^ Rank (*p* = 0.04), OR 2.96 (1.03–8.51), ref: Officer.

^9^ CESS (*p* = 0.02), OR 1.12 (1.01–1.21).

^10^ Education (*p* = 0.04), OR 0.59 (0.36–0.97), ref: Some college or more.

^11^ Sex (*p* = 0.004), OR 0.24 (0.10–0.64), ref: Female.

^12^ CESS (*p* = 0.01), OR 1.12 (1.02–1.23).

^13^ Rank (*p* = 0.02), OR 6.10 (1.43–26.12), ref: Officers.

^14^ Sex (*p* = 0.002), OR 0.20 (0.07–0.55), ref: Female.

AP, assessment period; CESS, Combat Exposure Severity Score; CI, confidence interval; LOC, loss of consciousness; MDD, major depressive disorder; mTBI, mild TBI; OR, odds ratio; PTSD, post-traumatic stress disorder; SSD, somatic symptom disorder; TBI, traumatic brain injury.

Using mTBI without LOC as a reference group, associations between mTBI with LOC and symptoms were analyzed as shown in Table 6. Those with mTBI with LOC had significantly increased odds of MDD in AP2 (aOR, 5.40; 95% CI, 1.12–26.36; *p* < 0.05) and SSD in AP1 (aOR, 8.13; 95% CI, 1.65–40.03; *p* < 0.01) and AP2 (aOR, 3.28; 95% CI, 1.07–10.01; *p* < 0.05).

**Table 6.** Unadjusted and Adjusted Risk of Self-Reported PTSD, MDD, and SSD Symptoms by Severity of TBI and Presence of LOC in mTBI (*n* = 157)

	Mild TBI with LOC (*n* = 79)		Moderate-to-severe TBI (*n* = 336)	
	Odds ratio (95% CI)			
	Unadjusted	Adjusted	Unadjusted	Adjusted
PTSD				
AP1	2.82 (0.72–11.04)	3.34 (0.80–13.86)^a^	0.46 (0.11–1.86)	0.41 (0.09–1.86)^a^
AP2	1.67 (0.73–3.84)	1.50 (0.63–3.59)^b,1^	0.85 (0.41–1.73)	0.88 (0.42–1.87)^b,1^
MDD				
AP1	2.09 (0.60–7.23)	2.33 (0.64–8.45)^a,2^	0.28 (0.07–1.07)	0.33 (0.08–1.34)^a,2^
AP2	5.51^*^ (1.17–26.02)	5.40^*^ (1.12–26.36) ^c^	3.46 (0.81–14.82)	3.61 (0.83–15.82)^c^
SSD				
AP1	6.15^*^ (1.32–28.72)	8.13^**^ (1.65–40.03) ^3,4^	2.80 (0.64–12.10)	3.00 (0.65–13.53)^3,4^
AP2	2.88 (0.97–8.50)	3.28^*^ (1.07–10.01) ^c,5^	1.07 (0.40–2.92)	0.85 (0.30–2.40)^c,5^

Reference group: mild TBI without LOC (*n* = 78).

Adjusted models include age, sex, combat stress score, military rank, marital status, number of past deployments, branch, education, and duty status as covariates.

^a^ Branch and rank not included.

^b^ Rank not included.

^c^ Branch not included.

^*^ *p* < 0.05, ^**^p < 0.01, ^***^p < 0.001.

Demographic variables significant in each model:

^1^ Age (*p* = 0.02), OR 1.06 (1.01–1.11).

^2^ CESS (*p* = 0.04), OR 1.28 (1.01–1.62).

^3^ Sex (*p* = 0.003), OR 0.09 (0.02–0.44), ref: Female.

^4^ CESS (*p* = 0.007), OR 1.26 (1.06–1.48).

^5^ Deployment (*p* = 0.03), OR 2.32 (1.06–5.05), ref: 2 or more deployments.

AP, assessment period; CESS, Combat Exposure Severity Score; CI, confidence interval; LOC, loss of consciousness; MDD, major depressive disorder; mTBI, mild TBI; OR, odds ratio; PTSD, post-traumatic stress disorder; SSD, somatic symptom disorder; TBI, traumatic brain injury.

## Discussion

Findings demonstrated that mTBI, not MS-TBI, is associated with increased risk for developing neuropsychiatric symptoms within the first year post-injury. Additionally, mTBI with LOC, not mTBI without LOC, increased the risk for PTSD, MDD, and SSD symptoms compared to non-TBI. Our findings dispute the notion that there is a positive association between TBI severity and neurocognitive outcomes.^25,26^ However, our findings support that those with mTBI tend to endorse significantly more symptoms, including those related to pain, self-perceived difficulties, and emotional distress, compared to those with MS-TBI.^28,40,57–62^ Additionally, our results are consistent with several studies demonstrating that PCL scores reported by mTBI subjects are higher than those reported by MS-TBI subjects, and that PTSD is more likely to occur in the context of mTBI.^63–65^

The precise reason why mTBI subjects present with increased symptoms in the early period post-injury is not fully understood. One interpretation is that, in contrast to MS-TBI which involves more prolonged LOC, mTBI may be associated with greater memory encoding of the traumatic event. This notion is consistent with a study that reported an inverse relationship between the duration of post-traumatic amnesia and severity of re-experiencing symptoms related to PTSD in patients with mTBI at the 3-month follow-up.^66^ In other words, the strength with which traumatic memories are encoded may correlate with PTSD symptom development.^67–70^

Similar to past studies, MS-TBI subjects had decreased odds of symptoms in AP1 compared to those without TBI, suggesting that MS-TBI may be protective against early-onset neuropsychiatric symptoms. For instance, a study found that MS-TBI patients, not mTBI patients, had fewer psychiatric diagnoses than their non-TBI counterparts.^71^ Another study reported that SMs had fewer self-reported neurobehavioral and post-traumatic stress symptoms after MS-TBI compared to mTBI.^72^

There are several interpretations for such a phenomenon. First, some MS-TBI patients may lack awareness and have impaired insight or apathy of their condition, whereas mTBI subjects may generally be more self-aware of their distress, leading to greater symptom reporting.^10,73,74^ Importantly, such a lack of awareness can negatively impact treatment and recovery post-TBI because of patients' underestimating of impairments and overestimating of functional abilities, leading to missed diagnoses.^75–77^ Other explanations for why MS-TBI patients may present with fewer symptoms in the early period include variations in post-injury factors, such as length of hospitalization, during which significant support is provided to ameliorate such symptomology.^78,79^ MS-TBI patients are more likely to require hospital intervention and intensive rehabilitation. Thus, these patients may not realize the extent of their non-physical impairments, given that the controlled hospital environment may limit the cognitive and social demands of everyday life.

Compared to the mTBI without LOC group, MS-TBI exhibited decreased PTSD symptoms, whereas mTBI with LOC showed increased PTSD symptoms, although not statistically significant. These findings may have relevance to the complex role of LOC on traumatic memories post-TBI. Given the current and previous findings that mTBI carries a higher risk of PTSD than severe TBI,^42^ it is conceivable that the longer duration of LOC, commonly observed in MS-TBI, may be protective against PTSD in the early period post-injury. One study, evaluating 47 SMs with moderate TBI who had no recollection of the event, postulated that the lack of intrusive memories and re-experiencing was a direct result of LOC-related amnesia.^38^

In contrast, the protective effects of LOC were less pronounced when comparing mTBI with or without LOC. mTBI subjects with LOC, not mTBI subjects without LOC, exhibited increased neuropsychiatric symptoms compared to subjects without TBI. This is consistent with past studies discussing LOC as an indicator of injury severity and that mTBI with LOC patients have worse neuropsychiatric outcomes compared to mTBI without LOC.^40,80^ It is possible that mTBI patients with LOC are still able to develop emotional reactions to traumatic events despite the lack of overt memory and conscious recall. For instance, studies suggested that those with mTBI and LOC may develop ”pseudo-memories” or collections of memory, despite being amnesic for the traumatic event.^81–83^ Such pseudo-memories can trigger declarative memory of the traumatic event by secondhand information, such as newspaper coverage, subjectively generated images, or other indirect stimuli. Another consideration is that certain persons with LOC may have some memory of the trauma leading up to the TBI and thus not be fully amnesic. This may be more common in those with relatively shorter duration of LOC and mTBI and hence contribute to the current findings. Yet, despite these possible explanations, the precise relationship between LOC and memory of the traumatic event is unclear, and thus further investigation is needed to unravel the neurological mechanisms of LOC and their effects on memory formation and neuropsychiatric symptom development.^84–86^

There are several limitations in the current study. First, although this study used a longitudinal study design to determine the association between TBI severity and development of symptoms, only two time periods in the first year post-injury were used for data analysis because of data availability. Second, there is an incomplete body of clinical information, such as clinician-determined psychiatric evaluations and pre-injury factors (i.e., lifetime history of TBIs and pre-existing psychiatric diagnoses) that may influence the onset and trajectory of symptom development. Third, TBI diagnoses were based on the DoD's classification of TBI severity using ICD-9 codes without specific information regarding injury mechanisms. The possible inaccuracy of categorizing TBI severity using ICD-9 codes has been described in past studies.^87,88^

Further, the timing of TBI relative to other physical trauma cannot be definitively known, though it is likely that such injuries occurred concurrently considering that SMs were evacuated because of polytrauma, including TBI. Fourth, additional follow-up data were not available to determine whether there were group differences in response to therapy. Finally, self-reported symptoms were used to categorize participants into either syndrome-present or -absent group rather than using a clinician-determined diagnosis. This approach may have resulted in some patients being misclassified because of differences in individual interpretation of questions and rating scales, and personal biases.

## Conclusion

We recommend that anyone exposed to trauma that could cause TBI be immediately professionally screened for such with thorough documentation of LOC. Further, though MS-TBI patients may report fewer symptoms in the early period after brain injury, symptom onset may be delayed, and thus long-term follow-up is recommended. Finally, the complex relationship between TBI severity and development of neuropsychiatric symptoms in injured SMs reinforce the need for a multi-disciplinary approach. Holistic and collaborative care approaches to the evaluation and treatment of coexisting symptom-based disorders are important in designing intervention strategies.^89–95^ Ultimately, TBI-related neuropsychiatric disorders constitute an imperative health burden in the military population, and a concerted effort is much warranted to address the devastating impact of TBI on military SMs and their families.

## Supplementary Material

Supplemental data

## Supplementary Material

Supplementary Data S3

Supplementary Table ST1

## Abbreviations Used

AIS: Abbreviated Injury Scale
APs: Assessment Periods
AP1: Assessment Period 1
AP2: Assessment Period 2
CESS: Combat Exposure Severity Score
CI: confidence interval
DoD: Department of Defense
DVBIC: Defense and Veterans Brain Injury Center
ICD-9: International Classification of Diseases, Ninth Revision
IQR: interquartile range
ISS: Injury Severity Score
LOC: loss of consciousness
MDD: major depressive disorder
mTBI: mild TBI
MS-TBI: moderate-to-severe TBI
PCL-C: PTSD Checklist, Civilian Version
PTSD: post-traumatic stress disorder
SMs: service members
SSD: somatic symptom disorder
TBI: traumatic brain injury
