# Supplementary material for: Association of Traumatic Brain Injury Severity and Self-Reported Neuropsychiatric Symptoms in Wounded Military Service Members
Source: Neurotrauma Rep. 2023 Jan 10;4(1):14–24. doi: 10.1089/neur.2022.0063 (PMC9886188; doi:10.1089/neur.2022.0063)
Supplement: Supplemental data [file Suppl_Data.docx]

**Supplementary Material**

**Supplementary Data S1.** Traumatic Brain Injury (TBI) Severity Classification Using Defense and Veterans Brain Injury Center (DVBIC) 2015 ICD-9 Diagnostic Codes.

**Supplementary Data S2.** Additional Methods

**Supplementary Data S3.** Combat Exposure Severity Score Survey Questions.

**Supplementary Table ST1.** Abbreviated Injury Scale (AIS) and Injury Severity Score (ISS).

**Supplementary Data S1.** Traumatic Brain Injury (TBI) Severity Classification Using Defense and Veterans Brain Injury Center (DVBIC) 2015 ICD-9 Diagnostic Codes

All subjects in the dataset had ICD-9 codes for their physical injuries. Any subject with a TBI-related ICD-9 code was categorized as part of the TBI group. Any subject without TBI-related ICD-9 codes was categorized as part of the non-TBI group. Thereafter, the severity of TBIs were classified as mild, moderate, or severe according to the DVBIC criteria.

Full document on ICD-9 codes can be accessed through this link: [**https://www.health.mil/Reference-Center/Publications/2015/12/01/Traumatic-Brain-Injury**](https://www.health.mil/Reference-Center/Publications/2015/12/01/Traumatic-Brain-Injury)

[m] - Concussion/Mild TBI is characterized by the following: Confused or disoriented state which lasts less than 24 hours; or loss of consciousness for up to 30 minutes; or memory loss lasting less than 24 hours. Excludes penetrating TBI. A CT scan is not indicated for most patients with a Mild TBI. If obtained, it is normal.

[M] - Moderate TBI is characterized by the following: Confused or disoriented state which lasts more than 24 hours; or loss of consciousness for more than 30 minutes, but less than 24 hours; or memory loss lasting greater than 24 hours but less than seven days; or meets criteria for Mild TBI except an abnormal CT scan is present. Excludes penetrating TBI. A structural brain imaging study may be normal or abnormal.

[S] - Severe TBI is characterized by the following: Confused or disoriented state which lasts more than 24 hours; or loss of consciousness for more than 24 hours; or memory loss for more than seven days. Excludes penetrating TBI. A structural brain imaging study may be normal but usually is abnormal.

[P] - Penetrating TBI, or open head injury, is characterized by the following: A head injury in which the scalp, skull and dura mater (the outer layer of the meninges) are penetrated. Penetrating injuries can be caused by high-velocity projectiles or objects of lower velocity such as knives, or bone fragments from a skull fracture that are driven into the brain.

[U] - Unclassifiable TBI

**Supplementary Data S2.** Additional Methods

**A. Assessment Periods (APs):**

APs were determined based on the distribution of data collected over time as described in our previous paper.^1^ Initial and follow-up data were generally collected before post-injury day 75 and after post-injury day 90 (Figure 1 from Soumoff et al. 2021).^1^ Very few data were collected between days 75 and 90 post-injury, and an insufficient number of SMs were surveyed frequently enough to create smaller divisions of APs.

**B. Neuropsychiatric Symptom Assessments:**

The PCL-C is a 17-item self-report measure of severity of each symptom of PTSD from the DSM-IV-TR over the prior month. ^2^ Responses were scored with a 5-point Likert scale ranging from 1 (*not at all)* to 5 (*extremely)*, with a possible total score ranging from 17 to 85. Similar to previous studies, ^1, 3^ the criteria for self-reported PTSD were a rating of 3 or higher on at least one symptom of intrusion, three symptoms of avoidance, and two symptoms of hyperarousal, with a total score of 50 or higher.

The PHQ-9 contains nine items inquiring about symptoms of a major depressive episode, each rated from 0 (*not at all)* to 3 (*nearly every day)* with possible total scores ranging from 0 to 27. ^4^ The criteria for self-reported MDD were a rating of two or higher on at least five of the symptoms, with at least one including depressed mood or anhedonia as previously described. ^1, 3-5^

The PHQ-15 contains 15 items assessing the severity of somatic symptoms, such as localized pain, nausea, or dizziness, each rated from 0 (*not bothered at all*) to 2 (*bothered a lot*) with possible total scores ranged from 0 to 30. High severity of somatic symptoms was defined as PHQ-15 >10, as described previously. ^1, 6^

**C. Statistical Analyses:**

Subject characteristics were converted to binary variables including education (high school or less vs. college or more), marital status (married vs. other), military branch (Army vs. other), military rank (enlisted vs. warrant or commissioned officer), duty status (active duty vs. other), and number of deployments (1 vs. $\geq$2). Age and CESS were included as continuous variables. Unadjusted odds ratio (ORs) and adjusted odds ratios (aORs) for TBI status (mTBI and MS-TBI) and severity of TBI based on the LOC status were reported with 95% confidence intervals (CIs). Initial comparisons between subjects’ characteristics and symptom severity were performed using bivariate methods, including correlation analysis and t-tests with or without Welch’s correction, where appropriate. Data were missing at random for all demographic variables, except for sex and age which did not have missing data, at levels ranging from 0.2% to 17%. All cases were used for the current analyses.

**Supplementary Data S3.** Combat Exposure Severity Score Survey Questions.

1a) Questioning detainees or prisoners (recent deployment)

1b) Questioning detainees or prisoners (previous deployment)

2a) Physical ambush or assault (recent deployment)

2b) Physical ambush or assault (previous deployment)

3a) Being shot at (recent deployment)

3b) Being shot at (previous deployment)

4a) Seeing, handling, or smelling dead bodies or body parts (recent deployment)

4b) Seeing, handling, or smelling dead bodies or body parts (previous deployment)

5a) Being in or witnessing an accident causing serious injury or death (recent deployment)

5b) Being in or witnessing an accident causing serious injury or death (previous deployment)

6a) Clearing/searching homes, buildings, caves or bunkers (recent deployment)

6b) Clearing/searching homes, buildings, caves or bunkers (previous deployment)

7a) Sexual assault or rape (recent deployment)

7b) Sexual assault or rape (previous deployment)

8a) Knowing someone seriously injured or killed (recent deployment)

8b) Knowing someone seriously injured or killed (previous deployment)

9a) Seeing dead or seriously injured Americans (recent deployment)

9b) Seeing dead or seriously injured Americans (previous deployment)

10a) Shooting or directly firing at others (recent deployment)

10b) Shooting or directly firing at others (previous deployment)

11a) Seeing innocent victims of war (recent deployment)

11b) Seeing innocent victims of war (previous deployment)

Combat Exposure Severity Score (maximum possible score = 11)

1 point assigned for each positive response for either recent or previous deployments, or both.

**Supplementary Table ST1.** Abbreviated Injury Scale (AIS) and Injury Severity Score (ISS)

|  | **All Subjects** | **Non-TBI** | **mTBI** | **MS-TBI** |
| --- | --- | --- | --- | --- |
|  | **Mean (SD)** | | | |
| **ISS** | 16.4 (10.5) | 14.0 (9.1) | **16.1 (8.8)*** | **22.2 (11.9)**^** |
| **AIS Body Region 1 – Head, neck, and C-spine** | 1.0 (1.5) | 0.2 (0.7) | **1.5 (0.6)**** | **2.7 (1.6) **^** |
| **AIS Body Region 2 – Face** | 0.5 (0.8) | 0.2 (0.5) | 0.3 (0.5) | **1.3 (0.9)**^** |
| **AIS Body Region 3 – Chest, thorax, T-spine** | 0.7 (1.4) | 0.6 (1.3) | 0.9 (1.4) | **0.9 (1.5)*** |
| **AIS Body Region 4 – Abdomen, L-spine** | 0.8 (1.4) | 0.8 (1.4) | 1.0 (1.4) | 0.7 (1.2) |
| **AIS Body Region 5 – Extremities – upper, lower, and pelvis** | 2.1 (1.4) | 2.3 (1.3) | 2.3 (1.4) | **1.6 (1.5)**^** |
| **AIS Body Region 6 – External - skin** | 1.1 (0.7) | 1.1 (0.7) | 1.0 (0.6) | 1.1 (0.6) |

mTBI: mild TBI, MS-TBI: moderate-severe TBI

***** p < 0.05 compared to non-TBI; ** p <0.001 compared to no TBI

^ p < 0.001 compared to mTBI

**References**

1. Soumoff, A.A., Clark, N.G., Spinks, E.A., Kemezis, P.A., Raiciulescu, S., Driscoll, M.Y., Kim, S.Y., Benedek, D.M. and Choi, K.H. (2021). Somatic symptom severity, not injury severity, predicts probable posttraumatic stress disorder and major depressive disorder in wounded service members. Journal of traumatic stress.

2. Blanchard, E.B., Jones-Alexander, J., Buckley, T.C. and Forneris, C.A. (1996). Psychometric properties of the PTSD Checklist (PCL). Behav Res Ther 34, 669-673.

3. Grieger, T.A., Cozza, S.J., Ursano, R.J., Hoge, C., Martinez, P.E., Engel, C.C. and Wain, H.J. (2006). Posttraumatic stress disorder and depression in battle-injured soldiers. Am J Psychiatry 163, 1777-1783; quiz 1860.

4. Kroenke, K., Spitzer, R.L. and Williams, J.B. (2001). The PHQ-9: validity of a brief depression severity measure. J Gen Intern Med 16, 606-613.

5. Spitzer, R.L., Kroenke, K. and Williams, J.B. (1999). Validation and utility of a self-report version of PRIME-MD: the PHQ primary care study. Primary Care Evaluation of Mental Disorders. Patient Health Questionnaire. JAMA 282, 1737-1744.

6. Kroenke, K., Spitzer, R.L. and Williams, J.B. (2002). The PHQ-15: validity of a new measure for evaluating the severity of somatic symptoms. Psychosom Med 64, 258-266.
